# Supplementary material for: A potential cost of evolving epibatidine resistance in poison frogs
Source: BMC Biol. 2023 Jun 28;21:144. doi: 10.1186/s12915-023-01637-8 (PMC10303791; doi:10.1186/s12915-023-01637-8)
Supplement: Supplementary file 2 — Additional file 2. Effects of reciprocal substitutions on ACh concentration-response curves (CRC) in the β2 subunit of human and dendrobatid frog, Epipedobates anthonyi. Data redrawn from Tarvin et al. [13], presented as mean ± SD. (A) A high ratio of β2 to α4 (1α:3β) of cRNA of the wild type human receptor subunits produces a monophasic CRC with a single EC50 indicating only high sensitivity (HS) binding sites (black curve: β2(FS) represents F106 and S108 in β2 subunit; n = 7). Introduction of the S108C substitution adds a low sensitivity (LS) binding site so that the CRC is now best fit with a biphasic curve reflecting both HS and LS sites (green curve: β2(FC), amino acid in bold indicates a substitution; n = 6). Further addition of F106L to S108C eliminates the LS sites, thus compensating for the effect of S108C [orange curve: β2(LC); n = 6]. (B) A low ratio of β2 to α4 (3α:1β) of the wild type human receptor subunits produces an ACh CRC shifted rightward and best fit with a monophasic curve with a shallow slope [black curve: β2(FS); n = 13]. Introduction of the S108C substitution shifted the curve further right (green curve: β2(FC); n = 6]. Addition of F106L to S108C partially compensates for the effect of S108C alone [orange curve: β2(LC); n = 5]. (C) When the ratio of injected human β subunit cRNA/α subunit cRNA is high, the nAChR stoichiometry is 2α:3β. However, with paucity of β subunits the stoichiometry shifts to 3α:2β. (D,E) Even with more extreme ratios of α and β subunits (1:7 and 7:1) and the introduction of the ancestral amino acids (FS, FC), there was no change in the CRC of Epipedobates anthonyi (n = 5-13). (F) The stoichiometry of frog nAChR receptors is unknown but we conjectured it is 2α:3β because they show a single kind of binding site (HS). This conjecture is noted by the question mark over the grey arrow. [file 12915_2023_1637_MOESM2_ESM.pdf]

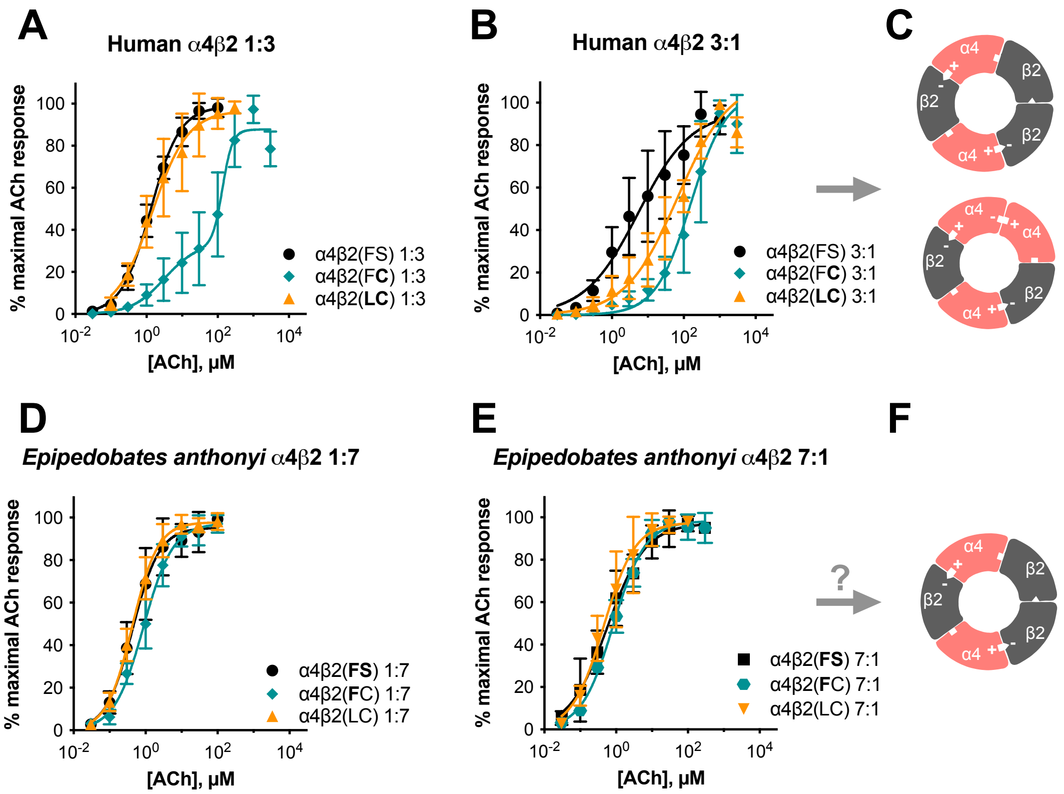

Additional file 2. Effects of reciprocal substitutions on ACh concentration-response curves (CRC) in the β2 subunit of human and dendrobatid frog, *Epipedobates anthonyi*.
Data redrawn from Tarvin et al.[1], presented as mean ± SD. (A) A high ratio of β2 to α4 (1α:3β) of cRNA of the wild type human receptor subunits produces a monophasic CRC with a single EC_{50} indicating only high sensitivity (HS) binding sites (black curve: β2(FS) represents F106 and S108 in β2 subunit; *n*= 7). Introduction of the S108C substitution adds a low sensitivity (LS) binding site so that the CRC is now best fit with a biphasic curve reflecting both HS and LS sites (green curve: β2(F**C**), amino acid in bold indicates a substitution; *n*= 6). Further addition of F106L to S108C eliminates the LS sites, thus compensating for the effect of S108C [orange curve: β2(**LC**); *n*= 6]. (B) A low ratio of β2 to α4 (3α:1β) of the wild type human receptor subunits produces an ACh CRC shifted rightward and best fit with a monophasic curve with a shallow slope [black curve: β2(FS); *n*= 13]. Introduction of the S108C substitution shifted the curve further right (green curve: β2(F**C**); *n*= 6]. Addition of F106L to S108C partially compensates for the effect of S108C alone [orange curve: β2(**LC**); *n*= 5]. (C) When the ratio of injected human β subunit cRNA/α subunit cRNA is high, the nAChR stoichiometry is 2α:3β. However, with paucity of β subunits the stoichiometry shifts to 3α:2β. (D,E) Even with more extreme ratios of α and β subunits (1:7 and 7:1) and the introduction of the ancestral amino acids (**FS**, **FC**), there was no change in the CRC of *Epipedobates anthonyi* (*n*= 5-13). (F) The stoichiometry of frog nAChR receptors is unknown but we conjectured it is 2α:3β because they show a single kind of binding site (HS). This conjecture is noted by the question mark over the grey arrow.

---

[1] Tarvin RD, Borghese CM, Sachs W, Santos JC, Lu Y, O'Connell LA, et al. Interacting amino acid replacements allow poison frogs to evolve epibatidine resistance. Science. 2017;357(6357):1261-6.
