# Supplementary material for: A potential cost of evolving epibatidine resistance in poison frogs
Source: BMC Biol. 2023 Jun 28;21:144. doi: 10.1186/s12915-023-01637-8 (PMC10303791; doi:10.1186/s12915-023-01637-8)
Supplement: Supplementary file 3 — Additional file 3. Accession numbers and names of species included in Fig. 1. The names of undefended species of poison frogs (Dendrobatidae) are in black and those of defended species are in blue. [file 12915_2023_1637_MOESM3_ESM.pdf]

Additional file 3. **Accession numbers and names of species included in Fig 1.** The names of undefended species of poison frogs (Dendrobatidae) are in black and those of defended species are in blue.

Subunit	Common name	Species	Accession number
β2	tunicate	*Ciona intestinalis*	NP_001265876.1
	sea lamprey	*Petromyzon marinus*	XP_032806233.1
	great white shark	*Charcharodon carcharias*	XP_041035646.1
	zebrafish	*Danio rerio*	XP_005169811.1
	human	*Homo sapiens*	CAD88996.1
	chicken	*Gallus gallus*	NP_990144.1
	Western clawed frog	*Xenopus tropicalis*	NP_001093684.1
	High Himalaya frog	*Nanorana parkeri*	XP_018425584.1
	grass frog	*Rana temporaria*	XP_040188232.1
	brilliant-thighed poison frog	*Allobates femoralis*	ATG31806.1
		Hyloxalus italoi	ATG31785.1
	strawberry poison frog	*Oophaga pumilio*	ATG71844.1
	Rio Santiago poison frog	*Excidobates captivus*	ATG31798.1
	golden poison frog	*Phyllobates terribilis*	ATG31810.1
	Ecuador poison frog	*Ameerega bilinguis*	ATG31790.1
		Silversotneia cf. gutturalis	ATG31801.1
		Leucostethus fugax	ATG31797.1
	Anthony's poison frog	*Epipedobates anthonyi*	ATG31796.1
α4	human	*Homo sapiens*	AAB40111.1
	Western clawed frog	*Xenopus tropicalis*	NP_001107315.1
	High Himalaya frog	*Nanorana parkeri*	XP_018415603.1
	Anthony's poison frog	*Epipedobates anthonyi*	ATG31830.1
