# Supplementary material for: A potential cost of evolving epibatidine resistance in poison frogs
Source: BMC Biol. 2023 Jun 28;21:144. doi: 10.1186/s12915-023-01637-8 (PMC10303791; doi:10.1186/s12915-023-01637-8)
Supplement: Supplementary file 4 — Additional file 4. F(DFn, DFd) and p values from one- and two population fittings to ACh concentration-response curves, calculated using the Extra sum-of-squares F test. A value of P < 0.10 means the preferred model is the “Two populations” (biphasic curve); P > 0.10 means the preferred model is the “One population” (monophasic curve). DFn, degree of freedom for the numerator of the F ratio, DFd is for the denominator. The amino acids between parentheses stand for the residues at locations 106 and 108, respectively; if there is a substitution, the letter is in bold font. [file 12915_2023_1637_MOESM4_ESM.pdf]

Additional File 4. **F(DFn, DFd) and p values from one- and two population fittings to ACh concentration-response curves, calculated using the Extra sum-of-squares F test.**

**Species**	**cRNA ratio**	**Receptor**	**Type of curve**	**F(DFn, DFd)**	***P* value**
Xenopus tropicalis	1:3	α4β2(FS)	One population	11.51 (2, 3)	0.04
		α4β2(F**C**)	One population	8.062 (2, 3)	0.06
		α4β2(**LC**)	One population	The other fit is ambiguous	
	7:1	α4β2(FS)	One population	5.998 (2, 3)	0.09
		α4β2(F**C**)	One population	The other fit is ambiguous	
		α4β2(**LC**)	-	-	-
Nanorana parkeri	1:3	α4β2(FS)	Two populations	The other fit is ambiguous	
		α4β2(F**C**)	Two populations	The other fit is ambiguous	
		α4β2(**LC**)	Two populations	The other fit is ambiguous	
	7:1	α4β2(FS)	Two populations	The other fit is ambiguous	
		α4β2(F**C**)	-	-	-
		α4β2(**LC**)	Two populations	The other fit is ambiguous	

A value of $P < 0.10$ means the preferred model is the “Two populations” (biphasic curve); $P > 0.10$ means the preferred model is the “One population” (monophasic curve). The amino acids between parentheses stand for the residues at locations 106 and 108, respectively; if there is a substitution, the letter is in bold font.

DFn: degree of freedom for the numerator of the F ratio

DFd: degree of freedom for the denominator of the F ratio
