# Supplementary material for: A potential cost of evolving epibatidine resistance in poison frogs
Source: BMC Biol. 2023 Jun 28;21:144. doi: 10.1186/s12915-023-01637-8 (PMC10303791; doi:10.1186/s12915-023-01637-8)
Supplement: Supplementary file 5 — Additional file 5. Representative tracings from ACh CRC from α4β2 nAChR of two species of non-dendrobatids Tracings from Xenopus tropicalis α4β2 nAChR with an α:β ratio of (A) 1:3 and (B) 7:1, and from Nanorana parkeri α4β2 nAChR with an α:β ratio of (C) 1:3 and (D) 7:1. ACh concentration is indicated above each peak, in μM. [file 12915_2023_1637_MOESM5_ESM.pdf]

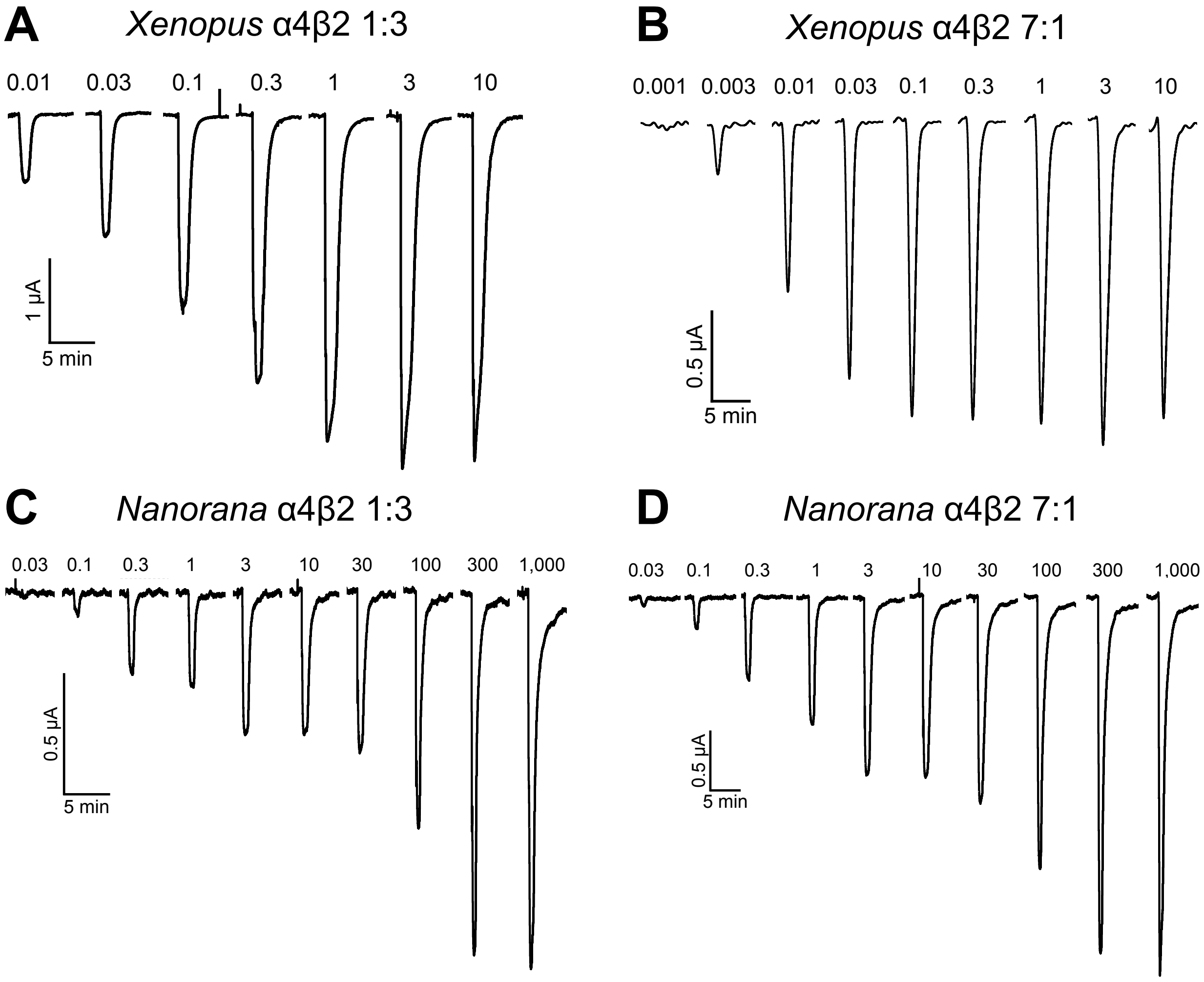

Additional file 5. Representative tracings from ACh CRC from α4β2 nAChR of two species of non-dendrobatids.

Tracings from *Xenopus tropicalis* α4β2 nAChR with an α:β cRNA ratio of 1:3 (panel A) and 7:1 (panel B), and from *Nanorana parkeri* α4β2 nAChR with an α:β cRNA ratio of 1:3 (panel C) and 7:1 (panel D). ACh concentration is indicated above each peak, in µM.
