# Supplementary material for: A potential cost of evolving epibatidine resistance in poison frogs
Source: BMC Biol. 2023 Jun 28;21:144. doi: 10.1186/s12915-023-01637-8 (PMC10303791; doi:10.1186/s12915-023-01637-8)
Supplement: Supplementary file 6 — Additional file 6. Maximal currents from α4β2 nAChR of two species of non-dendrobatids (A) Xenopus tropicalis (n = 10-39) (B) Nanorana parkeri (n = 9-20). The number over each bar indicates the total amount of cRNA (ng) injected per oocyte, while maintaining the α:β RNA ratio indicated. β2(FS) represents F106 and S108 in the β2 subunit. β2(FC) and β2(LC) indicates the residues present in position 106 and 108 in the β2 subunit, with the bold font indicating substitutions in the wild type background. The different amounts of cRNA injected precluded a complete statistical analysis of each data set, but we performed a two-way ANOVA on the Nanorana parkeri data set, followed by a Holm-Šídák analysis to correct for multiple comparisons (all conditions against all conditions). We only show the significant differences within each RNA ratio, as those oocytes were injected with the same total amount of cRNA. **p < 0.01, ****p < 0.0001. [file 12915_2023_1637_MOESM6_ESM.pdf]

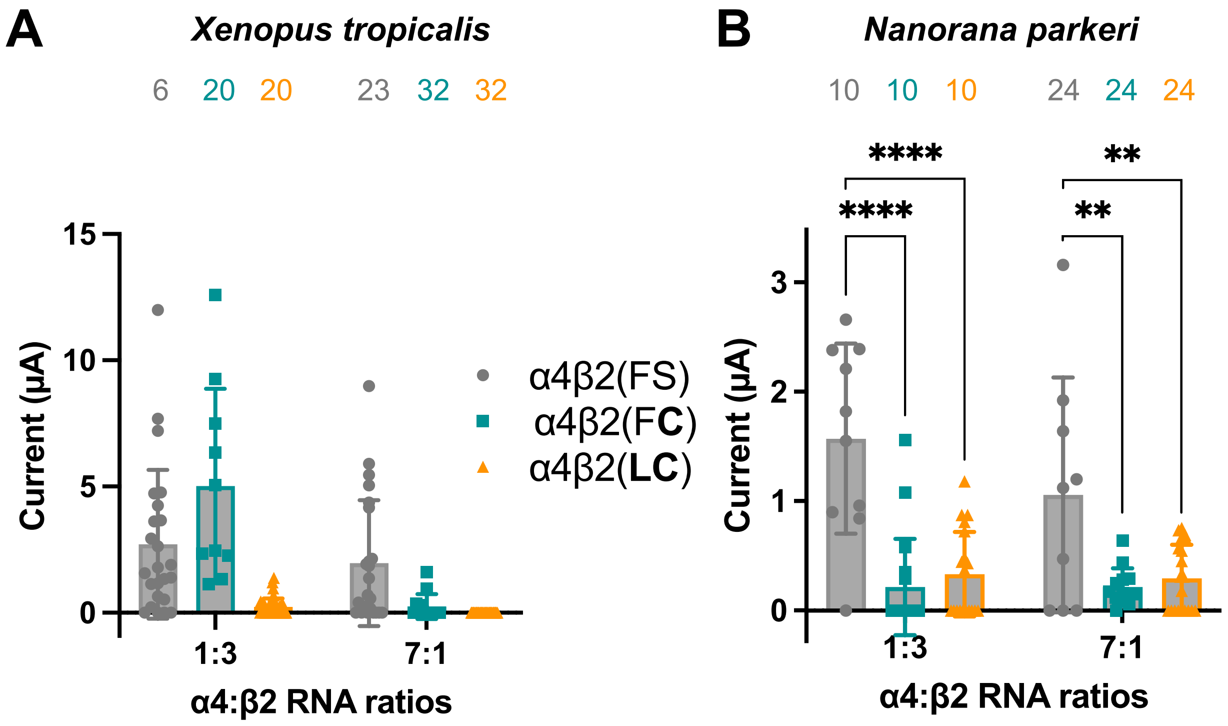

Additional File 6. Maximal currents from α4β2 nAChR of two species of non-dendrobatids. (A) *Xenopus tropicalis* (n= 10-39) (B) *Nanorana parkeri* (n= 9-20). The number over each bar indicates the total amount of cRNA (ng) injected per oocyte, while maintaining the α:β RNA ratio indicated. β2(FS) represents F106 and S108 in the β2 subunit. β2(F**C**) and β2(**LC**) indicate the residues present in position 106 and 108 in the β2 subunit, with the bold font indicating substitutions in the wild type background. The different amounts of cRNA injected precluded a complete statistical analysis of each data set, but we performed a two-way ANOVA on the *Nanorana parkeri* data set, followed by a Holm-Šídák analysis to correct for multiple comparisons (all conditions against all conditions). We only show the significant differences within each RNA ratio, as those oocytes were injected with the same total amount of cRNA. **p< 0.01, ****p< 0.0001.
